# The Proline-Rich Motif of the proDer p 3 Allergen Propeptide Is Crucial for Protease-Protease Interaction

**DOI:** 10.1371/journal.pone.0068014

**Published:** 2013-09-20

**Authors:** Marie-Eve Dumez, Julie Herman, Vincenzo Campisi, Ahlem Bouaziz, Frédéric Rosu, André Luxen, Isabel Vandenberghe, Edwin de Pauw, Jean-Marie Frère, André Matagne, Andy Chevigné, Moreno Galleni

**Affiliations:** 1 Macromolécules Biologiques, Centre for Protein Engineering, Université de Liège, Liège, Belgium; 2 Laboratoire de Rétrovirologie, Centre de Recherche Public de la Santé, Luxembourg, Luxembourg; 3 Laboratoire de spectrométrie de masse (L.S.M.), GIGA-R, Université de Liège, Liège, Belgium; 4 Centre de Recherches du cyclotron, Université de Liège, Liège, Belgium; 5 Laboratory for Protein Biochemistry and Protein Engineering, K.L. Ledeganckstraat 35B, Gent, Belgium; 6 Laboratoire d'enzymologie et repliement des protéines, Centre for Protein Engineering, Université de Liège, Liège, Belgium; University of Graz, Austria

## Abstract

The majority of proteases are synthesized in an inactive form, termed zymogen, which consists of a propeptide and a protease domain. The propeptide is commonly involved in the correct folding and specific inhibition of the enzyme. The propeptide of the house dust mite allergen Der p 3, NPILPASPNAT, contains a proline-rich motif (PRM), which is unusual for a trypsin-like protease. By truncating the propeptide or replacing one or all of the prolines in the non-glycosylated zymogen with alanine(s), we demonstrated that the full-length propeptide is not required for correct folding and thermal stability and that the PRM is important for the resistance of proDer p 3 to undesired proteolysis when the protein is expressed in *Pichia pastoris*. Additionally, we followed the maturation time course of proDer p 3 by coupling a quenched-flow assay to mass spectrometry analysis. This approach allowed to monitor the evolution of the different species and to determine the steady-state kinetic parameters for activation of the zymogen by the major allergen Der p 1. This experiment demonstrated that prolines 5 and 8 are crucial for proDer p 3-Der p 1 interaction and for activation of the zymogen.

## Introduction

Proteases control diverse processes including digestion, blood coagulation, immune reactions and apoptosis and are essential physiological factors [1]. However, their activities can be hazardous if uncontrolled [2]. Accordingly, studies describing the implication of endogenous or exogenous proteases in pathological processes such as Alzheimer's disease, cancer, malaria, AIDS and asthma have increased in recent years [3],[4],[5],[6],. Understanding the regulation of proteolytic activity is of great interest, particularly for the design and synthesis of new therapeutic molecules [2], [7].

For patients suffering from allergy (20–30% of the worldwide population), sensitization to house dust mite (HDM) allergens is a high risk factor for the development of asthma [9], [10]. Allergen groups 1, 3, 6 and 9 from *Dermatophagoides pteronyssinus* (*D. pteronyssinus*) and *D. farinae*, which contain cysteine (group 1, papaïn-like) and serine (group 3 trypsin, group 6 chymotrypsin and group 9 elastase-like) proteases, have been shown to be involved in the development and chronicity of allergy [11]. Indeed, these allergens induce IgE synthesis in allergic patients, and their proteolytic activity is involved in the targeting of epithelial cells and cells of the innate and adaptive immune systems [12]. These allergens can promote allergen penetration, IgE hyper-production and enhancement of the inflammatory process [13], [14], [15], [16].

Mites synthesize proteases as zymogens, which consist of an N-terminal propeptide of 6 to 82 amino acids and a protease domain of 220 to 232 residues corresponding to the active enzyme [11]. The most common function of propeptides is to inhibit the protease so that the enzyme-producing tissues are protected against non-specific degradation. After synthesis as an inactive zymogen, the propeptide is cleaved at precise times and locations by a specific intra- or inter-molecular process [2]. In the serine and cysteine protease families, the propeptide can also act as an intra-molecular chaperone, assisting with correct folding. Without this sequence, the enzyme remains blocked in a transitory inactive (molten globule) state, indicating that the propeptide is necessary for the late stage leading to the correctly folded proenzyme [17]. This process has been described in detail, including for α-lytic protease and subtilisin [18], [19], [20]. Recombinant expression of Der p 1 and Der p 3 in the absence of the propeptide sequence in *Escherichia coli* or *Saccharomyces cerevisiae* leads to incorrectly folded proteins with reduced IgE-binding reactivity, suggesting that the propeptides also act as intra-molecular chaperones [21], [22], [23], [24]. Most studies have focused on the role of the *D. pteronyssinus* major allergen Der p 1. However, our previous work demonstrated that Der p 3 exhibits a 50-fold higher catalytic efficiency and a less marked specificity than Der p 1 for residues in the P2 and P3 positions of substrates (Schechter and Berger's nomenclature [25]) [26], [27]. As a consequence, the list of known Der p 3 substrates involved in allergy (*e.g*., transmembrane protein junctions, the PAR-2 receptor of the lung epithelial cells and complement proteins C3 and C5) is likely not exhaustive [28], [29], [30].

Compared with other trypsin-like proteases, the propeptide of proDer p 3 has some distinct features. First, the absence of Lys or Arg in the P1 position results in inter-molecular activation mediated by Der p 1 [26]. Second, the 11-amino-acid propeptide of proDer p 3 contains 3 prolines that form a PxxPxxP motif (N**P_2_**IL**P_5_**AS**P_8_**NAT_11_). Such proline-rich motifs (PRMs) are known to induce conformational constraints that can protect peptides against non-specific proteolytic degradation [31]. Interestingly, PxxP motifs adopt a very stable structure, *i.e.*, a left-handed polyproline II (PPII) helix with three residues per turn in which the side chains are exposed on the surface of the helix. This arrangement provides an accessible interface for specific protein-protein interactions with little loss of entropy upon binding due to the rigidity of the structure [32], [33]. Therefore, the role of the proDer p 3 propeptide and, more specifically, of its PRM in the correct folding of the protein, in its activation mechanism and in the stability of the zymogen is of interest.

In this study, we report the involvement of the proDer p 3 propeptide in the formation of a correctly processed protein. Using quenched-flow coupled to mass spectrometry, we followed in real time the Der p 1-mediated activation of proDer p 3 mutants in which proline(s) were replaced by alanine(s). We demonstrated the crucial role played by the PRM and, in particular, of prolines 5 and 8 in the inter-molecular activation mechanism of proDer p 3.

## Materials and Methods

### Chemicals

Boc-IEGR-Methoxycoumarin acetate (MCA) and the cysteine protease inhibitor L-trans-epoxysuccinyl-leucylamido (4-guanidino) butane (E-64) were purchased from Bachem (Buttendorf, Switzerland). Unglycosylated recombinant mature Der p 1 was obtained from *Pichia pastoris* proDer p 1 as previously described [34]. Leucine enkephalin acetate salt hydrate was purchased from Sigma Aldrich (Saint-Louis, Missouri, USA).

### Expression of recombinant proDer p 3 zymogens in *Pichia pastoris*

The proDer p 3 mutants containing a single proline to alanine mutation are referred to as P2A, P5A and P8A. The zymogen in which all prolines are mutated to alanines is denoted as P-A. The proDer p 3 zymogens in which the 2, 5, 8 or 11 N-terminal amino acids are deleted are referred to as Δ1–2, Δ1–5, Δ1–8 or Δ1–11, respectively. All zymogens were constructed by PCR in which the *Pichia* codon-optimized N9Q proDer p 3 sequence was used as the template and primers introducing *EcoR* I and *Xba* I restriction sites were used, as previously described [26]. In these constructions, the N-glycosylation site in the propeptide (N9) was eliminated by substitution of asparagine by glutamine (N9Q). Briefly, after cloning into the pGEM-T Easy vector (Promega, Madison, USA) and DNA sequencing, proDer p 3 sequences were cloned into the pPICZαA vector downstream of the peptide signal of *Saccharomyces cerevisiae* factor α (Invitrogen, Groeningen, The Netherlands). After electroporation of the *P. pastoris* SMD1168 strain with the recombinant plasmids, transformants were selected on yeast extract peptone dextrose (YPD) medium containing zeocine (50 µg/ml) (Invitrogen). Expression of the zymogens (five clones per protein) was then tested by culturing in 100 ml buffered media with glycerol for yeast (BMGY) at 28°C until an A_600_ value of approximately 1 was reached. The cultures were centrifuged for 10 min at 5000 *g*. The pellets were resuspended in 100 ml buffered methanol-complex medium for yeast (BMMY, 0.5% methanol) for expression at 28°C for three days. The cultures were centrifuged at 10000 *g* for 10 min, and the supernatant was stored at −20°C. For each zymogen, the best producer was chosen after SDS-PAGE analysis, and expression of the proteins was performed for 48 hours in flasks (total volume of 1 L). The cultures were then centrifuged at 13000 *g* for 20 min, and supernatants containing the secreted proteins were stored at −20°C.

### Purification of recombinant proDer p 3 zymogens and Der p 3

Zymogens were purified from the supernatants of 1-L cultures as previously described [26] with slight modifications. Briefly, proteins were first purified by ion exchange chromatography with a Q Streamline exchanger (Amersham Biosciences, GE Healthcare, Uppsala, Sweden) and then a Q-HP Sepharose column (60 ml) (2.6×10 cm, Amersham Biosciences, GE Healthcare, Uppsala, Sweden). To completely eliminate the pigments present in the culture media, the flow-through fraction containing the zymogens was dialyzed at 4°C against 20 mM sodium acetate, pH 5.5 (buffer A), before purification on a CM-HP Sepharose column (25 ml) (1.6×10 cm, Amersham Biosciences, GE Healthcare, Uppsala, Sweden) equilibrated with buffer A. Bound proteins were progressively eluted with a linear gradient of buffer A containing 1 M NaCl over 10 column volumes. After SDS-PAGE analysis, fractions containing zymogens were pooled, dialyzed at 4°C against 20 mM ethanolamine/HCl, pH 9, and stored at −20°C. The concentration of zymogens was estimated by the BCA assay (Pierce, Rockford, USA).

After activation of 3 µM proDer p 3 by different concentrations of Der p 1 (20 nM for proDer p 3 and Δ1–2, 40 nM for proDer p 3 Δ1–5 and 340 nM for proDer p 3 Δ1–8) at 37°C for 90 min, the reaction was stopped by addition of 100 µM E-64. For each activation, mature Der p 3 was isolated by a fourth purification step on a 1-ml MonoQ column (0.5×5 cm, Amersham Biosciences, GE Healthcare, Uppsala, Sweden) equilibrated with 20 mM Tris-HCl, pH 8.5 (buffer B). Elution was performed with a linear gradient of buffer B containing 1 M NaCl over 10 column volumes. Fractions containing the Der p 3 activity were pooled and dialyzed against 20 mM ethanolamine/HCl, pH 9, before storage at −20°C.

### Fluorescence measurements

The intrinsic fluorescence of the purified proteins (4 µM) was recorded at 25°C in 20 mM ethanolamine/HCl, pH 9, on a Varian Cary Eclipse spectrofluorimeter using a scan rate of 350 nm min^−1^. The excitation and emission slit widths were 10 and 5 nm, respectively. The excitation wavelength was 280 nm, and emission spectra were recorded from 300 nm to 400 nm. The spectra were measured five times and averaged, and the fluorescence background of the buffer was subtracted.

For the heat-induced unfolding experiments, the temperature was increased from 20°C to 70°C at a rate of 0.5°C min^−1^. The fluorescence emission was monitored at 320 nm every 30 s. The transition curves were computed based on the assumption of a two-state model and analyzed according to the following equation [35]:
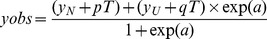
(1)where: 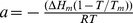; y_obs_ is the observed F signal at 320 nm at a given temperature; y_N_ and y_U_ represent the values of y_obs_ for the native and denatured states, respectively; p and q are the slopes of the pre- and post-transition baselines, respectively; R is the gas constant; T is the absolute temperature; T_m_ is the temperature of the mid-transition; and ΔH_m_ is the enthalpy value at T_m_. Because the transitions were irreversible, we considered only the (apparent) T_m_ value.

### N-terminal sequencing

The proteins were sequenced on an Applied Biosystems 476A protein sequencer (Applied Biosystems) using Edman degradation as previously described [26].

### Activation of proDer p 3 zymogens by Der p 1

The proDer p 3 zymogens (2.5 µM) were incubated at 37°C for various lengths of time with Der p 1 (30 nM) in PBS, pH 7.4, containing 5 mM DTT and 5 mM EDTA. Samples were analyzed by SDS-PAGE, and Der p 3 activity was determined. The samples were diluted 2000-fold in 50 mM polybuffer 1, pH 8.5 (mixture of 50 mM Tris, citrate, CAPS and potassium chloride) and the hydrolysis of 150 µM Boc-IEGR-MCA was monitored for 180 s at 37°C in a Perkin-Elmer LS 50 B instrument with excitation and emission wavelengths of 380 and 460 nm, respectively.

The activities of mature Der p 3 (200 nM) from activated proDer p 3 and from the activated Δ1–2, Δ1–5 and Δ1–8 proteins were monitored as described above and determined with the use of an MCA (Sigma, Saint-Louis, Missouri, USA) standard curve with concentrations ranging from 0 to 1.8 µM.

### Activation kinetics of proDer p 3 and P2A, P5A, P8A and P-A mutants

ProDer p 3 zymogens (12.5 nM) were activated at 37°C in the presence of increasing concentrations of Der p 1 (0–1.5 nM) in 50 mM polybuffer 2 (mixture of 50 mM Tris, phosphate, citrate, acetate and KCl containing 1 mM DTT and 1 mM EDTA, adjusted to pH 7.4). The Der p 3 enzymatic activity was monitored continuously by measuring the hydrolysis of 10 µM Boc-IEGR-MCA for 30 min.

Data were fitted to equation 2 and, the pseudo first-order rate constants (k_obs_) were plotted as a function of the Der p 1 concentration as previously described [26].
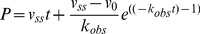
(2)where *P*, 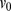, 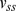 correspond respectively to the amount of MCA produced (µM), the initial rate for product release (µM/s) and the steady-state rate for product release (µM/s).

### Activation of proDer p 3 monitored by quenched-flow electrospray mass spectrometry

The proteins were desalted in 25 mM ammonium acetate, pH 7.4, with a Microcon system. In these experiments, the activating mixtures contained 16 µM proDer p 3 zymogens, 0.16 µM Der p 1 and 1.8 µM leucine enkephalin (internal standard) in 25 mM ammonium acetate, pH 7.4, with 1 mM DTT. The quenching solvent was an aqueous solution of 20% methanol and 0.8% acetic acid. These two solutions were continuously introduced via two independent syringe pumps in a mixing chamber at a 3 µl min^−1^ flow rate and injected into a Q-ToF Ultima Global electrospray mass spectrometer (Waters, Manchester, U.K.). The electrospray source was operated in positive ion mode (capillary voltage of 3 kV). Mass spectra were recorded with a speed of 20 min^−1^ and analyzed with MassLynx 4.1 software. An extracted ion current for each species (intensity vs. time) was reconstructed for quantification. The extracted intensities of the different charge states of a protein (m/z) were normalized with the intensity of the internal standard (leucine enkephalin) over time according to equation 3.
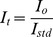
(3)where I_t_, I_o_ and I_std_ correspond to the normalized peak intensity of a defined charge state at time t, the peak intensity observed for this charge state and the peak intensity of the internal standard, respectively.

The normalized intensities corresponding to the different charged states of a protein were summed and the relative concentrations of the different proteins over time were then calculated according to equation 4.
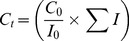
(4)where C_t_, C_0_, I_0_ and ΣI correspond respectively to the amount of protein at time t (µM), the initial concentration of zymogen (16 µM), the initial normalized peak intensity of zymogen and the sum of the normalized peak intensities of the different charge states of the protein to be considered, respectively.

For the P2A, P5A, P8A and P-A mutants, which exhibited truncated zymogen forms at the beginning of the reaction, I_0_ corresponded to the sum of the initial normalized peak intensities of these species.

### Nucleotide sequence accession number

The nucleotide sequence data encoding for N9Q proDer p 3, N9QS196A proDer p 3, P2A, P2AS196A, P5A, P5AS196A, P8A, P8AS196A, P-A, P-AS196A, Δ1–2, Δ1–2S196A, Δ1–5, Δ1–5S196A, Δ1–8, Δ1–8S196A, Δ1–11 and Δ1–11S196A have been deposited in the GenBank database under accession numbers KC879084, KC879085, KC879086, KC879087, KC879088, KC879089, KC879090, KC879091, KC879092, KC879093, KC879094, KC879095, KC879096, KC879097, KC879098, KC879099, KC8790100 and KC8790101, respectively.

## Results

### Role of the proDer p 3 propeptide in the production of a correctly processed protein

In our previous study, we showed that proDer p 3 expressed in *P. pastoris* is hyperglycosylated at the N_9_AT site of the propeptide. The use of the N9Q proDer p 3 mutant evidenced that glycoslylation is neither involved in the correct folding of the zymogen nor in inhibition of its enzymatic activity. However, glycosylation was very heterogeneous and it has been shown to decrease the zymogen activation rate [26]. To be independent of such a glycosylation regulation, all proteins expressed in this study contained the N9Q mutation. For all experiments, the N9Q proDer p 3 mutant was used as reference and termed proDer p 3.

To explore the role of the propeptide and, more specifically, of its proline residues in the recombinant expression, stability and activation of the proDer p 3 zymogen, the P2A, P5A, P8A and P-A mutants in which proline(s) are replaced by alanine(s) and the Δ1–2, Δ1–5, Δ1–8 and Δ1–11 proteins in which 2, 5, 8 and 11 residues of the propeptide are deleted were constructed and expressed in *P. pastoris* (Table 1). All proteins were secreted into the culture medium and purified to homogeneity from culture supernatants (1 L) to obtain between 6 and 15 mg of protein. For all zymogens, two additional residues (EF_−1_-) resulting from the *Eco* RI cloning site of the pPICZαA plasmid were observed as previously described (Table 1 and Table S1) [26].

**Table 1:** N-terminal sequences of proDer p 3 zymogens obtained after purification at 4°C.

Proteins	Expected and observed N-terminal sequences	Approximate (%)
**proDer p 3**	**NPILPASPQAT** ***IVGGEKALAG*** **…**	
4°C	EFNPILPASPQAT*IVGGEKALAG*	**100**
**P2A proDer p 3**	**NAILPASPQAT** ***IVGGEKALAG*** **…**	
4°C	EFNAILPASPQAT*IVGGEKALAG*	**100**
**P5A proDer p 3**	**NPILAASPQAT** ***IVGGEKALAG*** **…**	
4°C	EFNPILAASPQAT*IVGGEKALAG*	**100**
**P8A proDer p 3**	**NPILPASAQAT** ***IVGGEKALAG*** **…**	
4°C	EFNPILPASAQAT*IVGGEKALAG*	**100**
**P-A proDer p 3**	**NAILAASAQAT** ***IVGGEKALAG*** **…**	
4°C	EFNAILAASAQAT*IVGGEKALAG*	**8**
	ILAASAQAT*IVGGEKALAG*	**5**
	ASAQAT*IVGGEKALAG*	**9**
	SAQAT*IVGGEKALAG*	**42**
	AQAT*IVGGEKALAG*	**16**
	AT*IVGGEKALAG*	**14**
	*ALAGE*	6
**Δ1–2 proDer p 3**	**ILPASPQAT** ***IVGGEKALAG*** **…**	
4°C	EFILPASPQAT*IVGGEKALAG*	**88**
	SPQAT*IVGGEKALAG*	**3**
	*Not identified*	9
**Δ1–5 proDer p 3**	**ASPQAT** ***IVGGEKALAG*** **…**	
4°C	EFASPQAT*IVGGEKALAG*	**88**
	AT*IVGGEKALAG*	**8**
	*Not identified*	4
**Δ1–8 proDer p 3**	**QAT** ***IVGGEKALAG*** **…**	
4°C	EFQAT*IVGGEKALAG*	**24**
	EAEFQAT*IVGGEKALAG*	**74**
	*Not identified*	2
**Δ1–11 proDer p 3**	***IVGGEKALAG…***	
4°C	EAEF*IVGGEKALAG*	**94**
	EAEAEF*IVGGEKALAG*	**6**

All proteins contain the N9Q mutation, which abolishes N-glycosylation of the propeptide. The mature Der p 3 sequence is shown in italics. The EF N-terminal extension resulted from cloning.

The N-terminal sequences of proDer p 3 and of the P2A, Δ1–2, P5A and Δ1–5 zymogens were as expected despite the presence of small amounts (max 8%) of N-truncated forms (Table 1). By contrast, the P8A and P-A mutants were largely truncated in the propeptide and the protease domain, indicating a role for the prolines, especially proline 8, in resistance to non-specific degradation (Table S1). To reduce degradation, all proteins were purified at 4°C. Although the P-A mutant still exhibited truncation (Table 1), 94% corresponded to zymogen forms that could be activated by the Der p 1 protease.

The Δ1–11 protein, in which the complete propeptide of proDer p 3 is deleted and corresponds to “mature Der p 3”, presented two (**EA**EF_−1_-) or four additional residues (**EAEA**EF_−1_-) compared to other zymogens (Table 1). These residues arise from incomplete processing of the α-factor of the yeast *Saccharomyces cerevisiae* leader sequence, which is upstream of the proDer p 3 sequence in the pPICZα plasmid.

### Role of the proDer p 3 propeptide in the acquisition of the tridimensional structure of the zymogen

**Figure 1:**
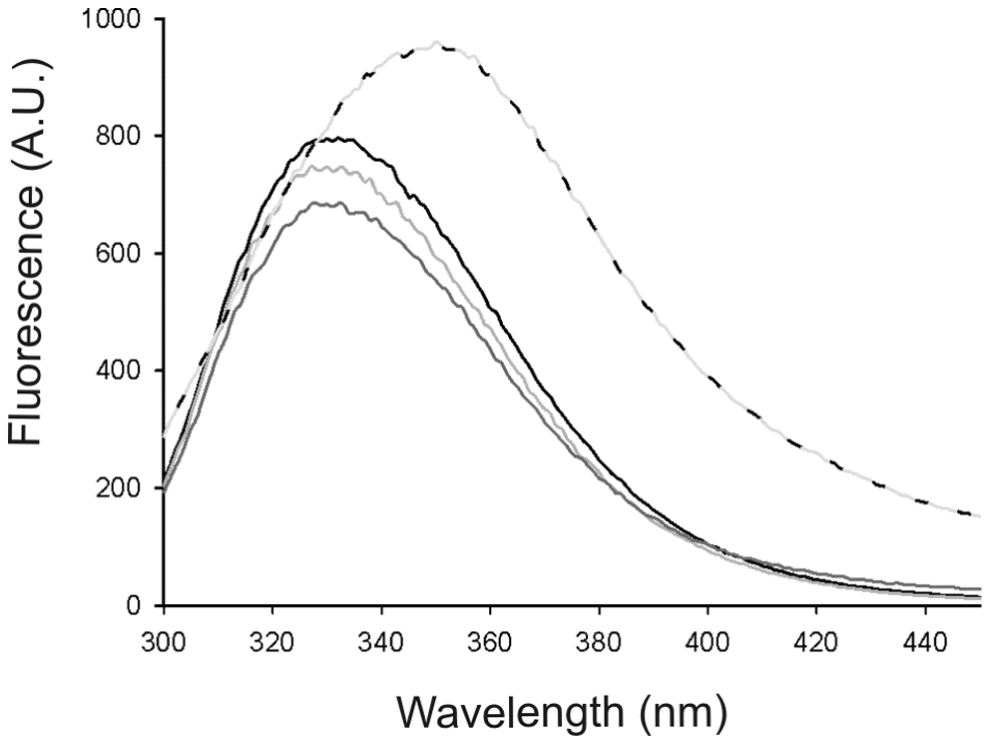
Intrinsic fluorescence emission spectra. Intrinsic fluorescence emission spectra of proDer p 3 (black), P-A (light gray), Δ1–11 (dark gray) and Der p 3 (dotted black-gray) in 20 mM ethanolamine/HCl, pH 9, at 25°C. A protein concentration of 4 µM and an excitation wavelength of 280 nm were used.

All proline mutants and deleted forms (four tryptophan residues) displayed intrinsic fluorescence spectra similar to that obtained for proDer p 3, with an emission maximum wavelength approximately 333 nm. This result indicates that all of the zymogens adopt the same global fold. The spectra of proDer p 3, P-A and Δ1–11 are shown in Fig. 1.

Following maturation of proDer p 3 by Der p 1, mature Der p 3 exhibited a red shift of the maximum emission wavelength to 350 nm. Interestingly, although the Δ1–11 protein does not contain the propeptide sequence, its fluorescence properties are “zymogen-like”.

The specific activities of the zymogens expressed in *P. pastoris* were measured with the Boc-IEGR-MCA substrate [26]. The activities were similar and ranged from 0.2 to 3% of the mature Der p 3 activity, although the activity of the Δ1–11 protein was only 0.01% (Table 2). A time-course SDS-PAGE analysis of the proDer p 3 mutants (3.9 µM) incubated at 37°C in 20 mM ethanolamine/HCl, pH 9, indicated that the P8A and P-A proteins were completely degraded within 24 to 48 hours instead of 48 to 96 hours for proDer p 3 and for the P2A and P5A mutants (data not shown).These results could be related to the slightly higher activity measured for P8A and P-A mutants (Table 2).

**Table 2:** k_cat_ values of 12.5 nM proDer p 3 zymogens determined with the use of 150 µM IEGR-MCA in 50 M polybuffer 1 at 37°C.

Zymogen	k_cat_ (min^−1^)
proDer p 3	10±1
P2A proDer p 3	11±0
P5A proDer p 3	5±0
P8A proDer p 3	40±3
P-A proDer p 3	103±3
Δ1–8 proDer p 3	23±3
Δ1–11 proDer p 3	0.25±0.03
Der p 3	3200±100

### Role of the proDer p 3 propeptide in the thermal stability of the protein

**Figure 2:**
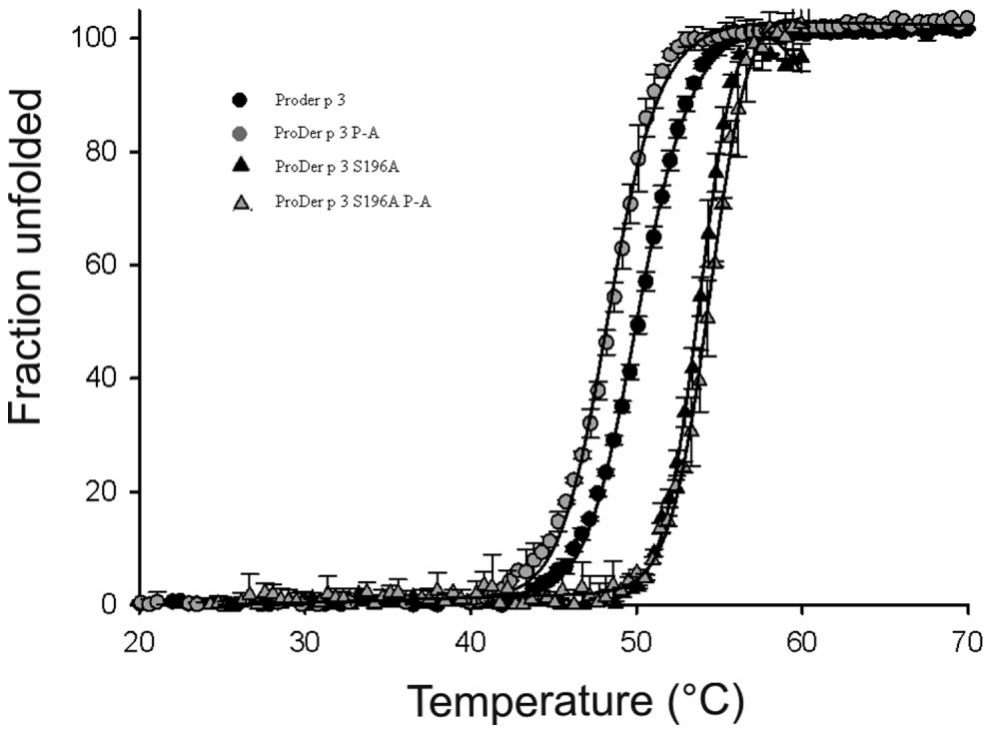
Heat-induced unfolding transitions monitored by fluorescence emission at 320 nm. The fractions of unfolded proDer p 3 wild type and mutants as a function of temperature.

Heat-induced denaturation (Fig. 2) was found to be irreversible. The transition curves obtained with all zymogens were similar and characteristic of a one-step unfolding mechanism. As shown in Table 3, the apparent mid-point values for the thermal unfolding of proDer p 3 and the proline mutants are similar but are slightly lower for the P8A and P-A zymogens, which is probably due to the higher proteolytic activity exhibited by these mutants.

**Table 3:** Apparent T_m_ values of the different proDer p 3 zymogens.

Zymogen	T_m_ (°C)
proDer p 3	49.5±0.1
P2A proDer p 3	49.1±0.1
P5A proDer p 3	49.4±0.1
P8A proDer p 3	48.7±0.1
P-A proDer p 3	48.1±0.1
S196A proDer p 3	54.2±0.1
P-AS196A proDer p 3	55.1±0.4

To test this hypothesis, the corresponding S196A mutants, in which the active serine (S196) is replaced by an alanine were studied (Fig. 2). All mutants exhibited the same intrinsic fluorescence spectra than their corresponding active forms (data not shown). The inactive mutants displayed a 5°C increase in their apparent T_m_ values, indicating that the enzymatic activity influences the stability of the proteins. Similar apparent T_m_ values (54–55°C, data not shown) were found with all the S196A proline mutants and the S196A deleted forms, indicating that the prolines of the propeptide should not be involved in the intrinsic thermal stability of the zymogen.

### Importance of the prolines in the propeptide of proDer p 3 for recognition by Der p 1

**Figure 3:**
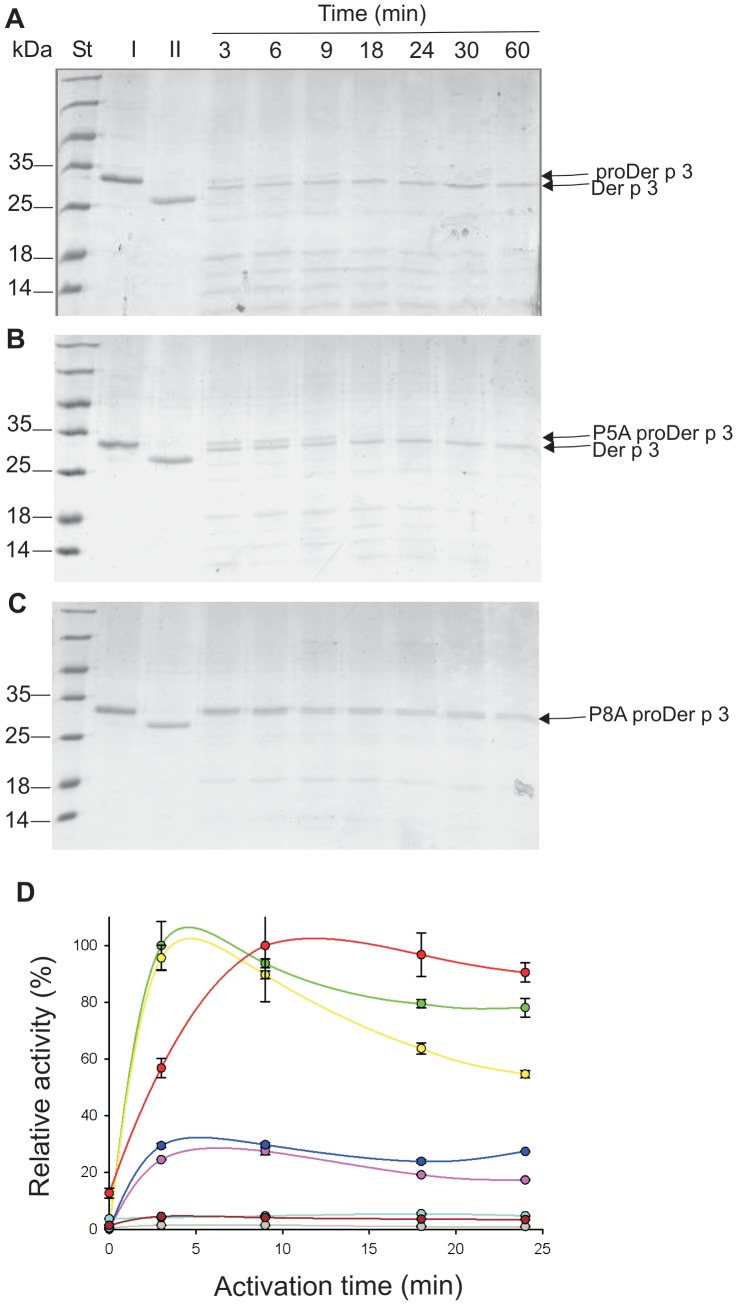
Inter-molecular activation of proDer p 3 zymogens by Der p 1. **A**: proDer p 3, **B**: P5A mutant and **C**: P8A mutant. SDS-PAGE (15%) analysis: 2.5 µM proDer p 3, P5A or P8A (lanes I) was incubated at 37°C in 50 mM PBS, pH 7.4, containing 5 mM DTT and 5 mM EDTA with 30 nM Der p 1. Lanes II: 2.2 µM Der p 1 as reference. The protein molecular mass marker (St) was from Fermentas. **D**: Der p 3 activities corresponding to the activations of (green) proDer p 3, (yellow) P2A, (red) Δ1–2, (pink) P5A, (blue) Δ1–5, (gray) P8A, (cyan) Δ1–8 or (dark red) P-A by 30 nM Der p 1 for increasing times of 0 to 24 min. Proteins were diluted 2000-fold, and Der p 3 activity was measured using IEGR-MCA as the substrate (150 µM).

As shown by SDS-PAGE analysis, the incubation of proDer p 3 in the presence of Der p 1 resulted in the total disappearance of the zymogen after 9 min (Fig. 3A). This maturation corresponds to the loss of the entire proDer p 3 propeptide in a one-step mechanism that is correlated with the increase in Der p 3 protease activity (Fig. 3D) [26]. After longer periods of time, the observed decrease in Der p 3 activity may be due to auto-hydrolysis at the NAK_115_ cleavage site of the enzyme, which is commonly observed in trypsin-like proteases [26], [36].

While activation of the P2A and Δ1–2 proteins yielded identical SDS-PAGE results (data not shown) and activity (Fig. 3D) profiles as those of proDer p 3, the P5A and Δ1–5 proteins displayed intermediate behaviors (Fig. 3B and 3D) with a maximum after 18 min that was only 25% of the expected activity.

The activation rates of the P8A, Δ1–8 and P-A proteins were drastically decreased. Indeed, the measured maximum Der p 3 activity was only approximately 5% proDer p 3 activation (Fig. 3D), and the appearance of mature Der p 3 was not observed by SDS-PAGE analysis (Fig. 3C). After the P8A and P-A mutants were incubated with Der p 1 for 6 hours, a truncated and inactive form of Der p 3 with an A_18_LAG- N-terminal sequence was observed.

**Figure 4:**
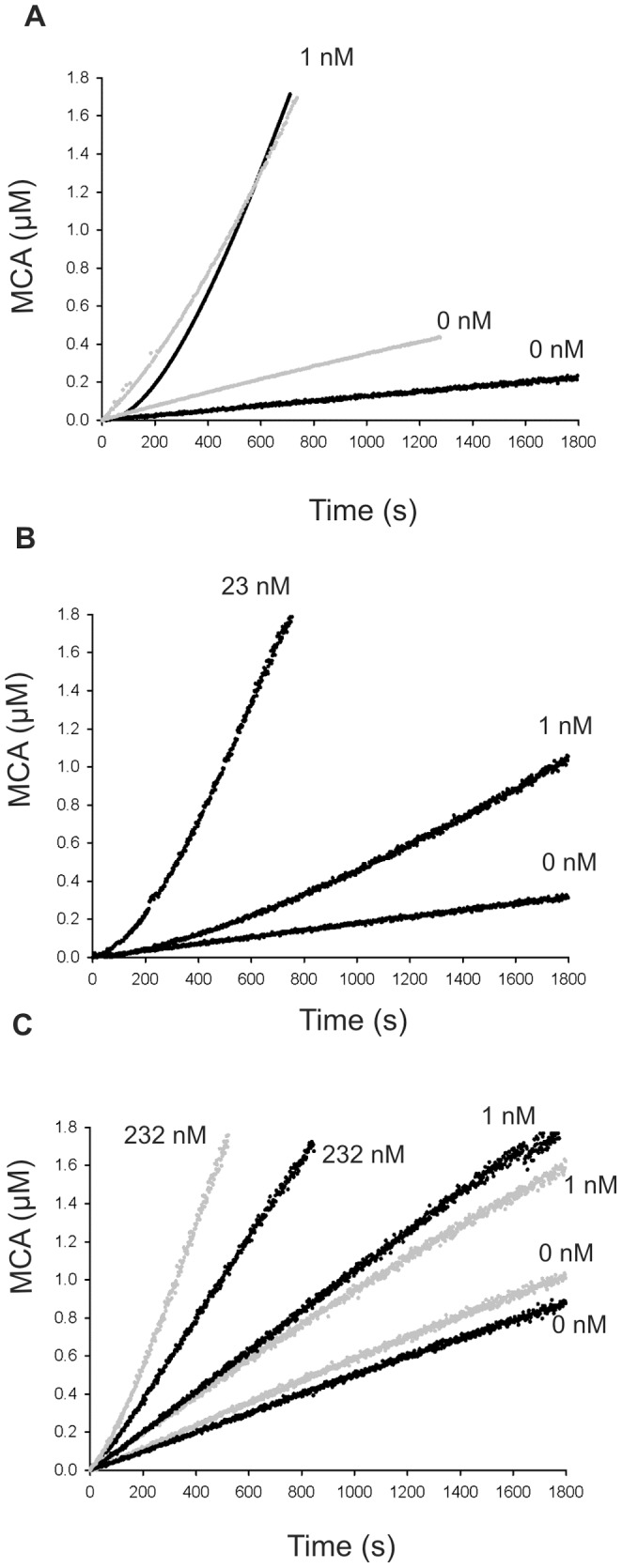
Continuous enzymatic assay for proDer p 3 maturation by Der p 1. **A**: proDer p 3 (black) and P2A (gray), **B**: P5A and **C**: P8A (black) and P-A (gray) mutants. Substrate hydrolysis (10 µM IEGR-MCA) versus time curves showing the activation of 12.5 nM zymogens by Der p 1 with the indicated concentrations in 50 mM polybuffer 2 at 37°C.

To increase the maturation rates, the zymogens were incubated with higher concentrations of Der p 1 for 30 min (Fig. 4), and Der p 3 activity was continuously monitored. In the absence of Der p 1, the activities of the zymogens were maintained constant with time, indicating that they cannot auto-activate themselves. Accordingly, a lack of auto-activation has been described for proDer p 3 [26]. However, the P8A and P-A proteins displayed higher basal enzymatic activities (Fig. 4C), which could be related to the N-terminal truncations of these mutants. The pseudo first-order rate constants, k_obs_ (Table 4) obtained according to equation 2, for the maturation kinetics of proDer p 3 and P2A by 1 nM Der p 1 were similar (Fig. 4A), whereas the activation rate of the P5A protein was lower (Fig. 4B). Moreover, at this activating concentration, the enzymatic activities of the P8A and P-A mutants were constant, indicating that they did not mature during the experimental timeframe. However, the slightly higher basal enzymatic activity detected in the presence of 1 nM Der p 1 remains unexplained (Fig. 4C). These results indicate that proline 8 and, to a lesser extent, proline 5 are essential for activation by Der p 1.

**Table 4:** Pseudo first order rate constants (k_obs_) for the maturation of the different proDer p 3 zymogens by 11 nM Der p 1.

Zymogen	k_obs_ (s^−1^)
WT proDer p 3	0.0035±0.0001
P2A proDer p 3	0.0029±0.0002
P5A proDer p 3	0.0011±0.0001

The use of Der p 1 at higher concentrations (23 and 232 nM) for the P5A (Fig. 4B) and P8A and P-A mutants (Fig. 4C), respectively, allowed the activation of the zymogens. Despite the high concentrations of Der p 1 needed to achieve these maturations, the neo-formed Der p 3 displayed an activity similar to that obtained upon maturation of proDer p 3.

Finally, proDer p 3 and the Δ1–2, Δ1–5 and Δ1–8 deleted proteins were totally activated by different concentrations of Der p 1 for 90 min. After total activation (as confirmed by SDS-PAGE), the corresponding active forms of the various zymogens were purified, and their specific activities were determined. The activities of the mature forms were similar (data not shown), confirming that they adopt the correct fold.

### Insights into the activation pathway of proDer p 3

The maturation of proDer p 3 and the proline mutants by Der p 1 was continuously monitored with a quenched-flow system coupled to a Q-ToF electrospray mass spectrometer. After desalting the proteins, proDer p 3 and Der p 1 were mixed in the presence of leucine enkephalin as an internal standard and mixed at a constant flow rate of 3 µl min^−1^ with co-solvent injected at the same rate. The mixture was continuously injected into the spectrometer, and mass spectra were recorded every 3 s during the maturation.

**Figure 5:**
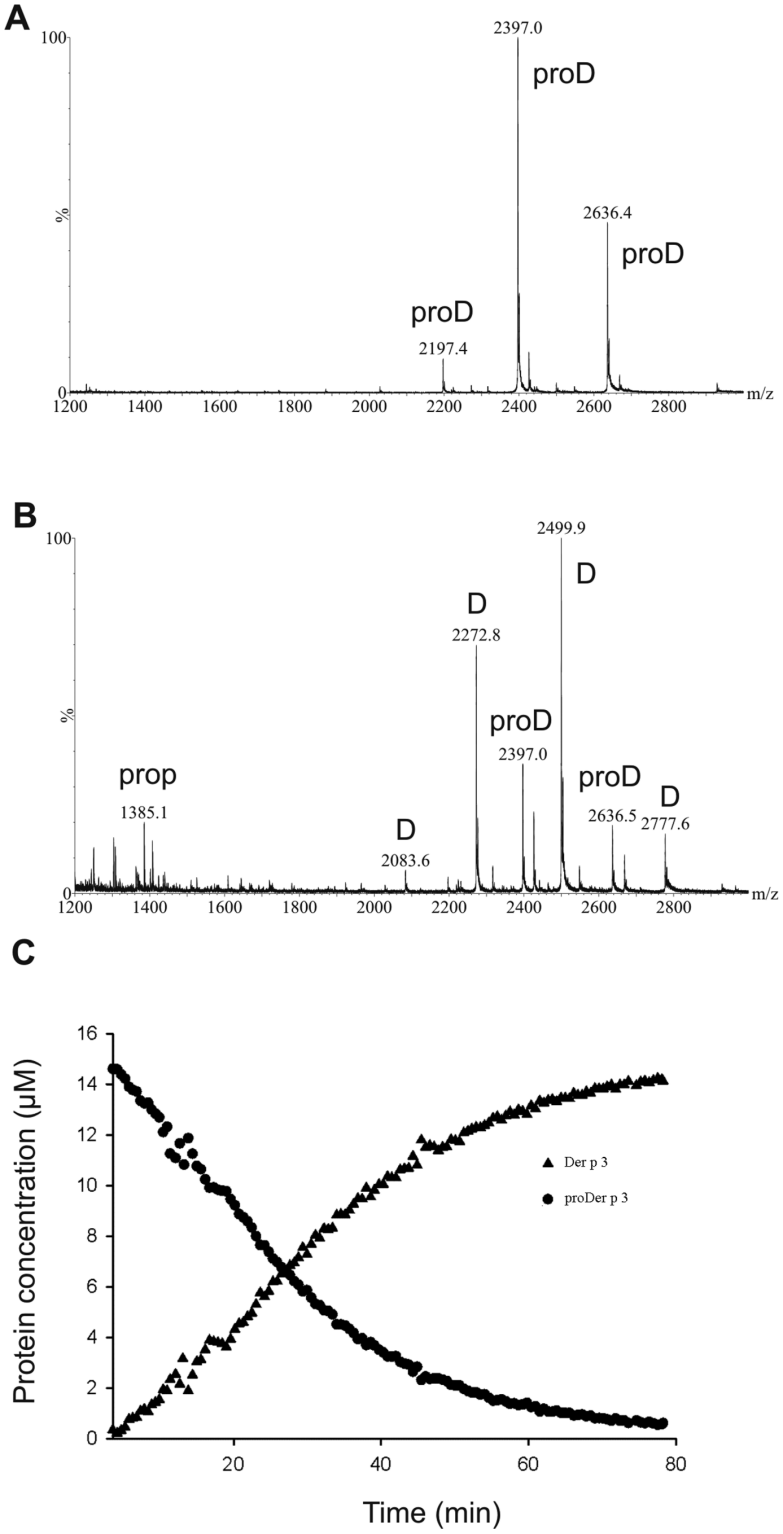
Continuous mass spectrometry assay for proDer p 3 maturation by Der p 1. Electrospray mass spectra (positive ion mode) for 16 µM proDer p 3 activation by 0.16 µM Der p 1 in 25 mM ammonium acetate, pH 7.4, containing 1 mM DTT, obtained after 5 min (**A**) and 40 min (**B**). ProD, D and prop refer to proDer p 3, Der p 3 and propeptide, respectively. **C**: The decrease in the proDer p 3 and the increase in the Der p 3 relative concentrations were calculated as described in the Material and Methods.

For the proDer p 3 activation, at the beginning of the reaction, ions with m/z values of 2197, 2397 and 2636, corresponding to [proDer p 3, mass: 26354 Da]^12+^, [proDer p 3]^11+^ and [proDer p 3]^10+^, respectively, were observed (Fig. 5A). These species decreased with time, whereas ions showing m/z values of 2083, 2273, 2500 and 2778, corresponding to [Der p 3, mass: 24987 Da]^12+^, [Der p 3]^11+^, [Der p 3]^10+^ and [Der p 3]^9+^, respectively, appeared together with the proDer p 3 propeptide (1385 Da) (Fig. 5B). In addition, low amounts of small unidentified fragments resulting from the non-specific hydrolysis of Der p 3 were also observed. For each ion, the peak intensity as a function of time was normalized against the peak intensity of the internal standard (leucine enkephalin, mass: 556 Da). The relative intensities of the different ions coming from one protein were summed, and the relative concentrations were calculated and are reported as a function of the activation time. Analysis of the kinetics shown in Figure 5C permitted the determination of an approximate Michaelis-Menten constant (K_m_) of Der p 1 for proDer p 3 of 3 to 6 µM and a specificity constant (k_cat_/K_m_) of 22 to 44 µM^−1^ s^−1^.

**Figure 6:**
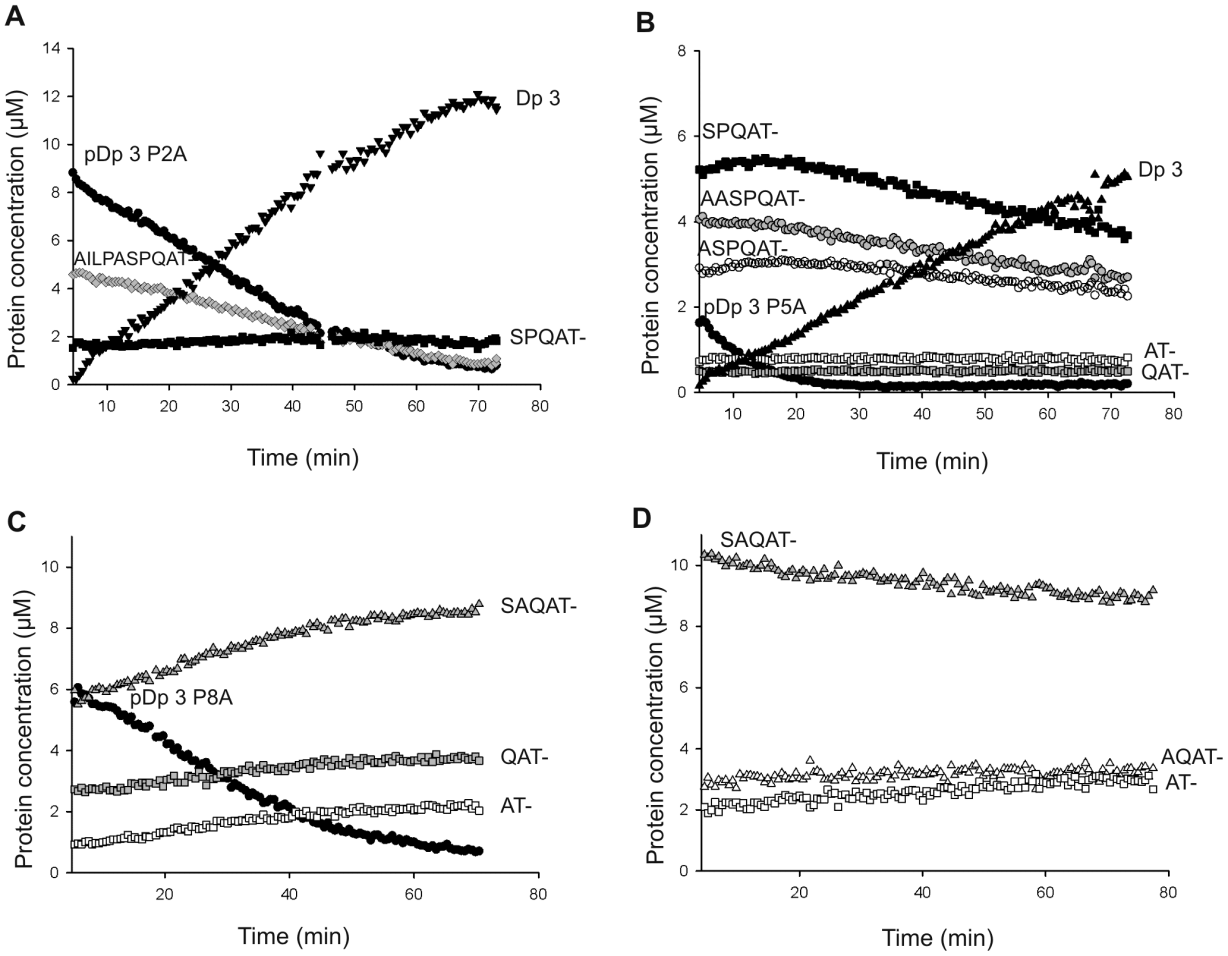
Continuous mass spectrometry assays for proDer p 3 mutants maturation by Der p 1. **A**: P2A, **B**: P5A, **C**: P8A and **D**: P-A mutants. Evolution of relative protein concentrations during the maturation of 16 µM zymogens by 0.16 µM Der p 1 in 25 mM ammonium acetate, pH 7.4, containing 1 mM DTT. The proteins were characterized as follows: entire proDer p 3 zymogen, rDer p 3, A_2_ILPAS- form, S_7_PQAT- form, A_5_ASPQA- form, A_6_SPQAT- form, QAT- form, AT- form, SAQAT- form and AQAT- form. Forms that are not described in Table 1 appeared during the desalting process.

Spontaneous hydrolysis of all proline mutants occurred upon desalting. Mass spectrometry permitted to independently monitor the different truncated forms during the activation process of proDer p 3 by Der p 1. For the P2A mutant, the entire zymogen and its truncated AILPASPQAT- form were activated whereas the SPQAT- form was unchanged (Fig. 6A). The intact form of P5A (EFNPILAASPQAT-) and its three truncated forms AASPQAT-, ASPQAT- and SPQAT- were processed into the native protein Der p 3 (IVGG-) (Fig. 6B). The processing rate was higher for the intact form. The QAT- and AT- forms were not processed at all. For the P8A mutant, the intact form disappeared; however, the processing yielded the truncated and inactive AT-, QAT- and SAQAT- forms rather than the native protein Der p 3 (Fig. 6C). Strikingly, and in contrast to the SPQAT form of the P5A mutant, the SAQAT form of the P8A protein was not further processed, which confirmed the importance of proline 8 in the activation reaction. For the P-A mutant, there was no intact zymogen at the beginning of the reaction, and the SAQAT-, AQAT- and AT- forms were not processed into Der p 3 (Fig. 6D). Notably, the proportions of active enzyme obtained for each zymogen maturation (Fig. 6) roughly correspond to those described in Fig. 3D.

## Discussion

When compared to other trypsin-like proteases, the Der p 3 propeptide exhibits some interesting and distinct features. In our previous study, we demonstrated that the absence of a DDDDK motif and the presence of a threonine at the C-terminus of the propeptide results in an unusual activation mechanism mediated by Der p 1 [26]. N-glycosylation at the N_9_AT- position, near the proDer p 3 cleavage site, decreases the maturation rate, as shown for Der p 1 [37]. The propeptide of Der p 3 also contains a PxxPxxP motif (N**P_2_**IL**P_5_**AS**P_8_**NAT_11_); the involvement of this motif in folding and activation by Der p 1 is the focus of the present study. To explore the role of these prolines, P2A, P5A, P8A and P-A mutants as well as the Δ1–2, Δ1–5, Δ1–8 and Δ1–11 proteins were constructed.

*P. pastoris* was used to produce all of the proteins as secreted forms. Examination of the purified preparations by N-terminal sequencing and electrospray mass spectrometry revealed the presence of different N-truncated forms for the P5A, P8A and P-A zymogens. Indeed, proline conformational constraints are known to induce structural resistance to non-specific degradations of the N-terminal X-proline sequences of several biological peptides, such as cytokines or growth factors [31]. Therefore, the prolines of the proDer p 3 prosequence are likely involved in the protection of the zymogen against undesired hydrolysis.

The presence of the additional residues EA and EAEA at the N-terminus of the Δ1–11 protein indicates that during its secretion in *P. pastoris*, the α-factor signal peptide is not completely cleaved by the dipeptidyl aminopeptidase. This result suggests that the presence of proDer p 3 propeptide is also important for correct processing (*i.e.*, elimination of the yeast signal peptide) of the zymogen during its secretion. In this case, the propeptide could be considered as a spacer situated between the signal peptide cleavage site and the core of the protein, promoting its correct processing.

Although proDer p 3 propeptide is involved in the production of a correctly processed protein and in the resistance to undesired proteolysis, it is not essential to obtain a correctly folded protein. Indeed, all proline mutants and deleted forms displayed the same fluorescence spectrum and exhibited similar residual activity. Moreover, after their total activation by Der p 1, the specific activities of the corresponding active forms were similar. Thus the proDer p 3 propeptide does not act as an intramolecular chaperone, as previously reported for other trypsin-like proteases [2]. In good agreement with our findings, Craik and collaborators [38], [39] demonstrated that the recombinant expression of an active trypsin in *E. coli* did not require the presence of a propeptide. More recently, the refolding of chemically denaturated bovine pancreatic trypsin was successfully performed without a propeptide [40].

The intrinsic fluorescence spectrum of Der p 3, obtained after activation of proDer p 3 by Der p 1, was characterized by a red shift which is also observed after maturation of trypsinogen in trypsin. It has been attributed to increased solvent exposure of W215 [41], [42]. Similarly, W214 could be more accessible to solvent in the mature form of the allergen Der p 3. Interestingly, the Δ1–11 protein in which the 11 amino acids of the proDer p 3 propeptide are deleted was characterized by a fluorescence spectrum and a proteolytic activity similar to the zymogen forms. These results suggest that the addition of four to six residues, as found at the N-terminus of the Δ1–11 protein, is sufficient to maintain Der p 3 in an inactive and “zymogen-like” conformation. It reiterates that the neo-formed N-terminal I12 is necessary to obtain an active conformation of Der p 3. These results can be compared to mechanism by which trypsinogen is activated to trypsin, in which the neo-formed N-terminal I16 forms a salt bridge and hydrophobic interactions with D194 that trigger formation of the so-called “activation domain” containing the S1 binding pocket and the oxyanion hole [43], [44].

The proline residues of the propeptide do not seem to be involved in the intrinsic thermal stability of the proenzyme. However, because the S196A mutants exhibit significantly higher apparent T_m_ (54–55°C instead of 48–49.5°C), denaturation of the proenzyme is likely accompanied by autolysis, as observed during the folding experiments of other proteases [40]. This phenomenon was more observed for the P8A and P-A mutants which display a slightly higher proteolytic activity than the other proDer p 3 zymogens.

As previously described, incubation of proDer p 3 with Der p 1 resulted in the loss of the proDer p 3 propeptide in a one-step mechanism, which was correlated with an increase in Der p 3 activity [26]. This was followed by a decrease in activity resulting from the autolysis of Der p 3. Under these conditions, the absence of proline 2 did not have major consequences because the behaviors of the P2A and Δ1–2 zymogens were similar to that of proDer p 3. However, mutation of proline 5 or 8 partially or totally impaired the maturation of proDer p 3, respectively. The truncated form of Der p 3 (A_18_LAG-), which exhibits identical apparent molecular weight to Der p 3 on SDS-PAGE analysis, was identified after six hours activation of P8A and P-A. This form was previously observed during the incubation of proDer p 3 with Der p 3 and it corresponds to the auto-hydrolysis of proDer p 3 after lysine 17 [26]. The nearly complete absence of increase of the activity after incubation of the P8A and P-A mutants with Der p 1 is due to a lack of activation and a partial auto-hydrolysis of zymogens. For the activation of the P5A mutant by Der p 1, although SDS-PAGE analysis indicates that the zymogen is fully matured, a low Der p 3 activity (25%) was observed. This is probably due to a combination of this auto-hydrolysis phenomenon and a reduced activation rate of the zymogen. Indeed, the increase of its activation rate by using a higher concentration of Der p 1, was sole sufficient to promote the maturation way leading to obtaining a fully active enzyme (100%).

The quenched-flow coupled to mass spectrometry method appeared to be useful to determine the time-course of an enzymatic process such as proteolysis but also to compare the catalytic efficiencies for the hydrolysis of a complex protein sample, like for P5A, P8A and P-A mutants which contain a mixture of species. Approximate catalytic parameters for the activation of proDer p 3 by Der p 1 (K_m_ of 3–6 µM and k_cat_/K_m_ of 22–44 µM^−1^ s^−1^) were determined with this method. Although the P2A and P5A mutants were activated into Der p 3 during the experiment, the maturation process of the latter was not complete. Interestingly, the concentration of Der p 3, as measured by mass spectrometry during the maturation process of the P5A mutant, corresponded to the activity measured by the enzymatic maturation test (25%). The P8A and P-A zymogens were not activated during the time course of the experiment, and only truncated forms appeared. Despite its slow maturation kinetics, the SPQAT- protein was processed, whereas the processing of the SAQAT- form was not detected under the conditions of the mass spectrometry experiments.

First, these experiments indicate that prolines 5 and 8 of the proDer p 3 propeptide are likely to be involved in the recognition and/or presentation of the zymogen to Der p 1, with a major contribution of proline 8. Usually, the major determinants for the protease specificity are the P4 to P1 positions of the substrate. Thus, proline 5, which is seven residues N-terminally located from the cleavage site (*i.e.* P7 position), is likely involved in the proper presentation of the propeptide to the cysteine protease. The specificity profile of Der p 1 highlights its preference for a proline at the P4 position of the substrate, which corresponds to proline 8 in the proDer p 3 propeptide [27].

Second, the activation kinetics of the deleted forms of proDer p 3 (Δ1–2, Δ1–5 and Δ1–8) were similar to those observed for their proline mutant counterparts (P2A, P5A and P8A), indicating that the amino acids surrounding the prolines and those that are N-terminally located to proline 5 in the propeptide seem to play a minor role in this processing.

In proteins, PRMs behave as common recognition sites for specific interactions. Indeed, the side chains of the superficial PPII helices are exposed to the solvent, and their structural rigidity reduces the entropy loss upon binding [33]. Conversely, the PRM-binding domains are often characterized by exposed aromatic side chains that can interact with the prolines of PRMs [32]. The P_5_ASP_8_ motif seems to be crucial for the Der p 1-proDer p 3 interaction. Interestingly, the Der p 1 protease catalytic groove is characterized by a hydrophobic surface that contains a conserved solvent-exposed aromatic cluster, which is likely involved in the interactions between the PRM of proDer p 3 and Der p 1 [45], [46].

Consequently, the conformation adopted by the propeptide and, in particular, the configuration of prolines 5 and 8 could be critical in the activation process and could determine the activation rate of Der p 1. Notably, propeptides in which the same proline residues are replaced by alanines or that are significantly shortened also favor abnormal truncations that further impair activation by Der p 1 or increase sensitivity to autolysis after K17.

In summary, we demonstrated that the proDer p 3 propeptide does not control the folding of proDer p 3 but is involved in the correct processing of the enzyme. Although prolines 5 and 8 do not greatly influence the thermal stability of the protein, these residues protect proDer p 3 against undesired hydrolysis and play a major role in maturation by the Der p 1 cysteine protease.

## Supporting Information

Table S1 N-terminal sequences of proDer p 3 zymogens obtained after purification at room temperature (RT). All proteins contain the N9Q mutation, which abolishes N-glycosylation of the propeptide. The mature Der p 3 sequence is shown in italics. The EF N-terminal extension resulted from cloning.
(DOC)
