# Supplementary material for: The Proline-Rich Motif of the proDer p 3 Allergen Propeptide Is Crucial for Protease-Protease Interaction
Source: PLoS One. 2013 Sep 20;8(9):e68014. doi: 10.1371/journal.pone.0068014 (PMC3779199; doi:10.1371/journal.pone.0068014)
Supplement: Table S1 — N-terminal sequences of proDer p 3 zymogens obtained after purification at room temperature (RT). All proteins contain the N9Q mutation, which abolishes N-glycosylation of the propeptide. The mature Der p 3 sequence is shown in italics. The EF N-terminal extension resulted from cloning. (DOC) [file pone.0068014.s001.doc]

**Table S1.** N-terminal sequences of proDer p 3 zymogens obtained after purification at room temperature (RT). All proteins contain the N9Q mutation, which abolishes N-glycosylation of the propeptide. The mature Der p 3 sequence is shown in italics. The EF N-terminal extension resulted from cloning.

| Proteins | Expected (bold) and observed N-terminal sequences |  |
| --- | --- | --- |
|  |  | Approximate % |
| **proDer p 3**  RT | **NPILPASPQAT*IVGGEKALAG*…**  EFNPILPASPQAT*IVGGEKALAG* | **100** |
| **P2A proDer p 3**  RT | **NAILPASPQAT*IVGGEKALAG*…**  EFNAILPASPQAT*IVGGEKALAG* | **100** |
| **P5A proDer p 3**  RT | **NPILAASPQAT*IVGGEKALAG*…**  EFNPILAASPQAT*IVGGEKALAG* | **100** |
| **P8A proDer p 3**  RT | **NPILPASAQAT*IVGGEKALAG*…**  SAQAT*IVGGEKALAG*  AT*IVGGEKALAG*  *ALAGE*  *Not identified* | **34**  **27**  38  1 |
| **P-A proDer p 3**  RT | **NAILAASAQAT*IVGGEKALAG*…**  SAQAT*IVGGEKALAG*  AQAT*IVGGEKALAG*  *ALAGE*  *Not identified* | **30**  **19**  40  11 |
